# Shape-Controlled Synthesis of Au Nanostructures Using EDTA Tetrasodium Salt and Their Photothermal Therapy Applications

**DOI:** 10.3390/nano8040252

**Published:** 2018-04-18

**Authors:** Youngjin Jang, Nohyun Lee, Jeong Hyun Kim, Yong Il Park, Yuanzhe Piao

**Affiliations:** 1 Schulich Faculty of Chemistry, Technion-Israel Institute of Technology, Haifa 3200003, Israel; youngjin@technion.ac.il; 2 School of Advanced Materials Engineering, Kookmin University, Seoul 02707, Korea; nohyunlee@kookmin.ac.kr; 3 Center for Nanoparticle Research, Institute for Basic Science, and School of Chemical and Biological Engineering, Seoul National University, Seoul 151-742, Korea; jhkim113@snu.ac.kr; 4 School of Chemical Engineering, Chonnam National University, Gwangju 61186, Korea; 5 Graduate School of Convergence Science and Technology & Advanced Institutes of Convergence Technology, Seoul National University, Suwon 16229, Korea

**Keywords:** gold, nanostructure, EDTA tetrasodium salt, photothermal therapy

## Abstract

Tuning the optical properties of Au nanostructures is of paramount importance for scientific interest and has a wide variety of applications. Since the surface plasmon resonance properties of Au nanostructures can be readily adjusted by changing their shape, many approaches for preparing Au nanostructures with various shapes have been reported to date. However, complicated steps or the addition of several reagents would be required to achieve shape control of Au nanostructures. The present work describes a facile and effective shape-controlled synthesis of Au nanostructures and their photothermal therapy applications. The preparation procedure involved the reaction of HAuCl_4_ and ethylenediaminetetraacetic acid (EDTA) tetrasodium salt, which acted as a reducing agent and ligand, at room temperature without the need for any toxic reagent or additives. The morphology control from spheres to branched forms and nanowire networks was easily achieved by varying the EDTA concentration. Detailed investigations revealed that the four carboxylic groups of the EDTA tetrasodium salt are essential for effective growth and stabilization. The produced Au nanowire networks exhibited a broad absorption band in the near-infrared (NIR) region, thereby showing efficient cancer therapeutic performance by inducing the selective photothermal destruction of cancerous glioblastoma cells (U87MG) under NIR irradiation.

## 1. Introduction

To date, metal nanostructures have attracted a great deal of attention because of their intriguing electronic, optical, and catalytic properties [1,2,3,4,5,6,7]. In the past few decades, Au nanostructures of various shapes, including nanospheres [8,9,10], nanorods [11,12,13,14,15,16,17,18,19], nanowires [20,21,22,23,24,25,26], polyhedrons [27,28,29,30,31,32], nanoplates [33,34,35], nanoshells [36,37,38], and branched forms [39,40,41,42,43,44,45,46,47,48,49], have been extensively explored because of their shape-dependent surface plasmon resonance properties [50,51,52,53]. The fascinating surface plasmon resonance properties of Au nanostructures enable their effective implementation in a wide variety of biomedical applications, such as cancer therapy, bio-imaging, biological sensing, and diagnostics [54,55,56,57,58]. Biological targets (e.g., protein and DNA) are recognized as changes in the absorption of the functionalized Au nanostructures and an efficient bio-imaging using Au nanostructures is achieved by their distinctive interactions with light. Photothermal therapy is one of the major therapeutic methods that requires near-infrared (NIR) light, which shows maximum penetration depths in tissues and has a low toxicity for normal cells, so that cancer cells can be killed by heat generation [56,59,60,61,62,63,64,65,66,67,68,69,70]. To obtain Au nanostructures exhibiting optical properties active in the NIR region, intricate methods including multiple steps, many reagents, or long reaction times have been applied. For example, a seed-mediated growth approach has been widely used to produce anisotropic Au nanostructures, such as nanorods, which require steps for the formation of Au seeds and their growth. In addition, a method using two (or more) appropriate capping reagents with different binding affinities has been developed to obtain kinetic control of the growth rates on various crystal planes, resulting in the formation of non-spherical Au nanostructures. Therefore, the development of Au nanostructure preparation that yields NIR absorption is of prime importance for the successful extension to practical applications.

Recently, it was reported that ethylenediaminetetraacetic acid (EDTA), a well-known metal chelating ligand, can act as a reducing agent [71,72,73]. However, to the best of our knowledge, the morphology control of Au nanostructures by applying EDTA agents has not been attempted. Therefore, this is pioneering work on the preparation of Au nanocrystals exhibiting NIR activities using EDTA agents at room temperature.

Herein we present a facile and effective shape-controlled synthesis of colloidal Au nanostructures at room temperature and their effective performance for photothermal therapy. This simple procedure was achieved by reacting HAuCl_4_ and EDTA tetrasodium salt, exhibiting a dual function as a reductant and a stabilizing agent, without any toxic compounds such as cetyltriethylammonium bromide or additional agents (e.g., superhydride). The morphology of the Au nanostructures was readily controlled by adjusting the molar ratio of the EDTA tetrasodium salt to the HAuCl_4_ precursor. Reducing the concentration of the EDTA tetrasodium salt led to the formation of Au nanowire networks that are active in the NIR regime. Control experiments using several kinds of EDTA agents revealed that the four deprotonated carboxylic groups of EDTA tetrasodium salt play a key role as stabilizing agents and in effective growth control. The Au nanowire networks showed an effective and selective photothermal therapeutic effect on cancerous glioblastoma cells (U87MG) under NIR irradiation at 980 nm.

## 2. Materials and Methods 

### 2.1. Materials

EDTA, EDTA disodium salt, and EDTA tetrasodium salt were purchased from Samchun Chemicals (Seoul, Korea). Tetrachloroaurate trihydrate (HAuCl_4_·3H_2_O) was purchased from Strem Chemicals, Inc. (Newburyport, MA, USA). Methoxy poly(ethylene glycol)sulfhydryl (mPEG-SH, M_w_ = 5000) was purchased from SunBio Corp. (Anyang, Korea). Water deionized by a Nano Pure System (Barnstead, Thermo Fisher Scientific, Waltham, MA, USA) was used. The chemicals used for the preparation of the solutions were purchased at the highest grade possible, and used without further purification.

### 2.2. Shape-Controlled Synthesis of Au Nanostructures

To synthesize Au nanowires with a network structure, 15 mg of EDTA tetrasodium salt was injected into 7 mL of deionized water at room temperature, and the mixture solution was stirred for a few minutes to ensure complete dissolution of the salt. Further, 0.1 mL of 0.1 M HAuCl_4_ was added to the aqueous solution containing the EDTA tetrasodium salt at room temperature, and the resulting solution was vigorously stirred for 1 h. After the reaction, the mixture was washed with water by centrifugation at 14,000 rpm for 10 min. To synthesize the branched Au nanoparticles, the molar ratio of HAuCl_4_ to EDTA tetrasodium salt was changed to 1:6, and other conditions were kept constant. To synthesize the spherical Au nanoparticles, the molar ratio of HAuCl_4_ to EDTA tetrasodium salt was increased to 1:8. 

To stabilize the Au nanowire networks, an mPEG-SH aqueous solution prepared by dissolving 40 mg of mPEG-SH in 2 mL of deionized water was injected into the mixture solution and further stirred for 30 min. The resulting solution was washed with water by centrifugation at 14,000 rpm for 15 min.

### 2.3. In Vitro Cytotoxicity against U87MG Cells

U87MG human glioblastoma cells were grown as monolayer cultures in a 100-mm dish and subcultured 3 times a week at 37 °C in an atmosphere of 5% CO_2_ and 100% relative humidity. For the in vitro cytotoxicity assay, cells at a logarithmic growth phase were detached and plated (0.2 mL per well) in 96-well flat-bottomed microplates at a density of 10,000 cells per well, which were then left for 1–2 days at 37 °C to resume exponential growth. After washing the cells with phosphate buffered saline (PBS), 0.1 mL of the culture medium, with various concentrations of Au nanowire networks coated with mPEG-SH ligand, was added to the wells in quintuplicate. For the control wells, the same volume of culture medium was included in each experiment. Following 24 h of continuous exposure to the Au nanowire networks under 5% CO_2_ atmosphere at 37 °C, cell survival was assessed using the (3-(4,5-dimethylthiazol-2-yl)-2,5-diphenyltetrazolium bromide (MTT) cell proliferation assay.

### 2.4. In Vitro Photothermal Therapy

The U87MG human glioblastoma cells were plated in a confocal dish and allowed to grow on the coverslip. Additionally, 150 μg Au/mL of the Au nanowire networks coated with mPEG-SH ligand were incubated with the cells for 24 h. The unbound Au nanowire networks were rinsed with the PBS buffer, and the cells were immersed in the culture medium.

Subsequently, a 980-nm continuous wave (CW) diode laser was used for irradiation and was focused to a 1-mm-diameter spot on the sample (power density: 38.2 W/cm^2^). The cells were irradiated for 10 min. The control cells without the Au nanowire networks with laser irradiation were also tested. Subsequently, the cells were stained with a few drops of 0.4% trypan blue to test viability. The dead cells were stained blue, while the live cells remained clear. After staining, the cells were rinsed with the PBS buffer and immersed in the culture medium for bright-field imaging.

### 2.5. Characterization

Transmission electron microscopy (TEM) and high-resolution TEM (HR-TEM) images were acquired using a JEOL EM-2010 microscope (JEOL, Tokyo, Japan) at an accelerating voltage of 200 kV. The powder X-ray diffraction (XRD) pattern was obtained by a Rigaku D/Max-3C diffractometer (Cu Kα radiation, λ = 0.15418 nm, Rigaku Co. Ltd., Tokyo, Japan). The UV-Vis absorption spectra were acquired by a Jasco V-570-type spectrophotometer (Jasco SLM-468, Tokyo, Japan). Elemental analysis was performed by inductively coupled plasma-atomic emission spectroscopy (ICP-AES) using an ICPS-7500 spectrometer (Shimadzu, Kyoto, Japan). The MTT cell proliferation assay was performed by an ELx808TM absorbance microplate reader (Biotek Instruments Inc., Winooski, VT, USA).

## 3. Results and Discussion

In general, colloidal synthesis of Au nanostructures requires reductants and capping agents. In the current procedure, the Au nanostructures were readily synthesized by reacting HAuCl_4_ and EDTA tetrasodium salt in an aqueous solution at room temperature. The EDTA tetrasodium salt played a dual role as a reducing agent and as a ligand, thus allowing for simple synthesis without any additives. After the reaction, the products were washed with water to remove any byproducts and unreacted reagents. More details are provided in the experimental section. 

**Figure 1:**
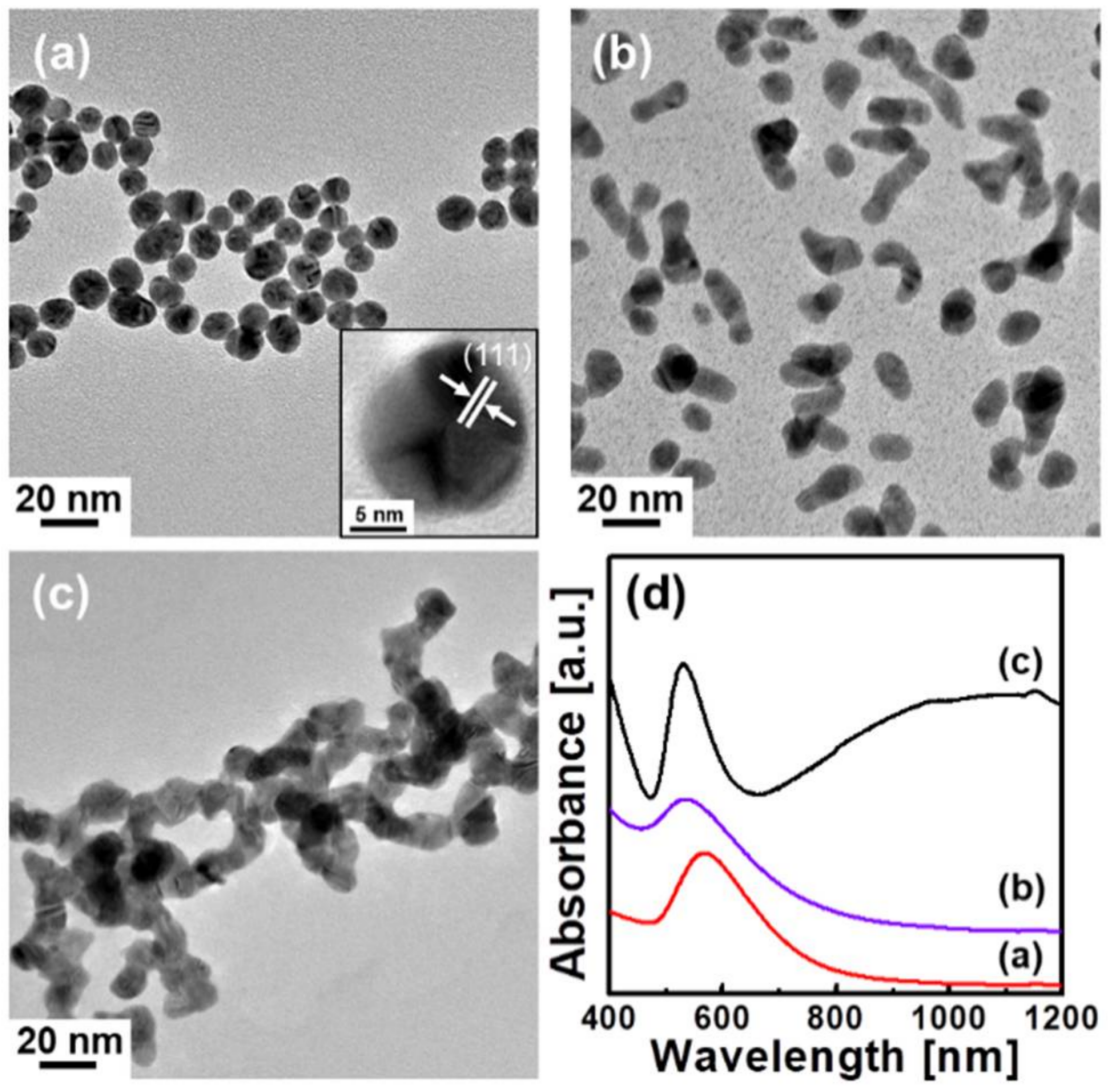
Transmission electron microscopy (TEM) images of Au nanostructures prepared using different molar ratios of HAuCl_4_ to ethylenediaminetetraacetic acid (EDTA) tetrasodium salt: (**a**) 1:8, the inset is the high-resolution TEM (HR-TEM) image of panel (**a**); (**b**) 1:6; (**c**) 1:4; (**d**) Absorption spectra of Au nanostructures in panels (**a**–**c**).

**Scheme 1:**
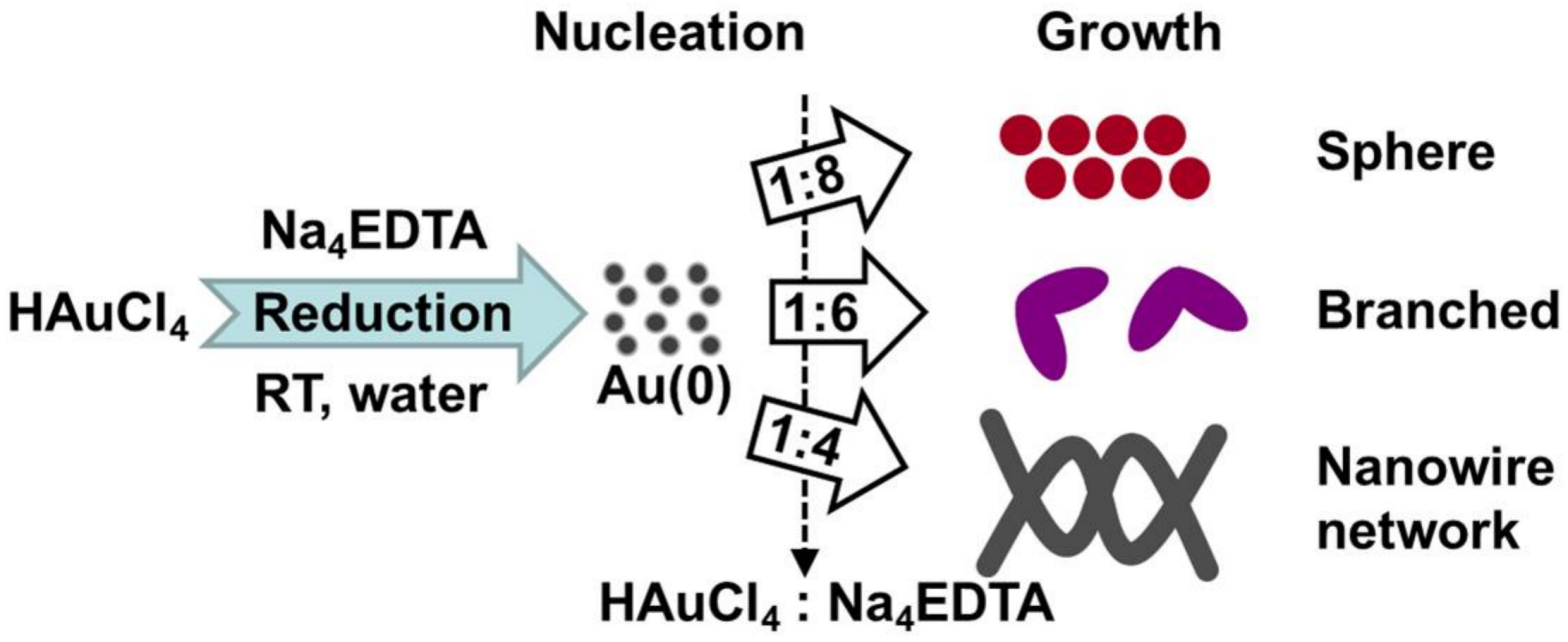
Schematic illustration of morphology control by adjusting the molar ratio of HAuCl_4_ to EDTA tetrasodium salt.

Figure 1a shows a representative TEM image of the Au nanostructures prepared using a 1:8 molar ratio of HAuCl_4_ to EDTA tetrasodium salt. The image presents the formation of spherical Au nanocrystals with an average diameter of 11 nm. A HR-TEM image is shown in the inset of Figure 1a, indicating the polycrystallinity of the Au nanocrystals with pronounced lattice fringes of 0.235 nm, corresponding to the (111) planes of a face-centered-cubic (fcc) Au crystal structure. Decreasing the molar ratio of HAuCl_4_ to EDTA tetrasodium salt induced shape control, resulting in branched forms (see Figure 1b) at 1:6 and nanowire networks (see Figure 1c) at 1:4. When the molar ratio was below 1:3, severely aggregated Au particles were observed because of the insufficient capping agent. Based on these observations, the formation of the Au nanostructures is summarized in Scheme 1, illustrating that the morphology of the Au nanostructures is easily controlled by adjusting the EDTA tetrasodium salt concentration. High concentrations of EDTA tetrasodium salt produced only spherical Au nanocrystals. Decreasing the molar ratio of HAuCl_4_ to EDTA tetrasodium salt resulted in the formation of non-spherical Au nanostructures, such as branched forms and nanowire networks. 

The absorption spectra of Au nanostructures, with three kinds of morphologies, are presented in Figure 1d, clearly displaying the shape-dependent surface plasmon resonance behaviors. Spherical Au nanocrystals (see red curve (a) in Figure 1d) exhibit one absorption peak at 570 nm, resulting from the transverse mode, while the transverse band becomes broader in the branched Au nanostructures (see purple curve (b) in Figure 1d). The most intriguing activity was observed when using a 1:4 molar ratio of HAuCl_4_ to EDTA tetrasodium salt (see black curve (c) in Figure 1d). Two distinct optical behaviors were identified in the absorption spectrum of the Au nanowire networks. The peak at 530 nm is attributed to transverse surface plasmon resonance (TSPR), and a broad absorption band over the NIR spectra regime was observed, owing to the longitudinal surface plasmon resonance (LSPR).

**Figure 2:**
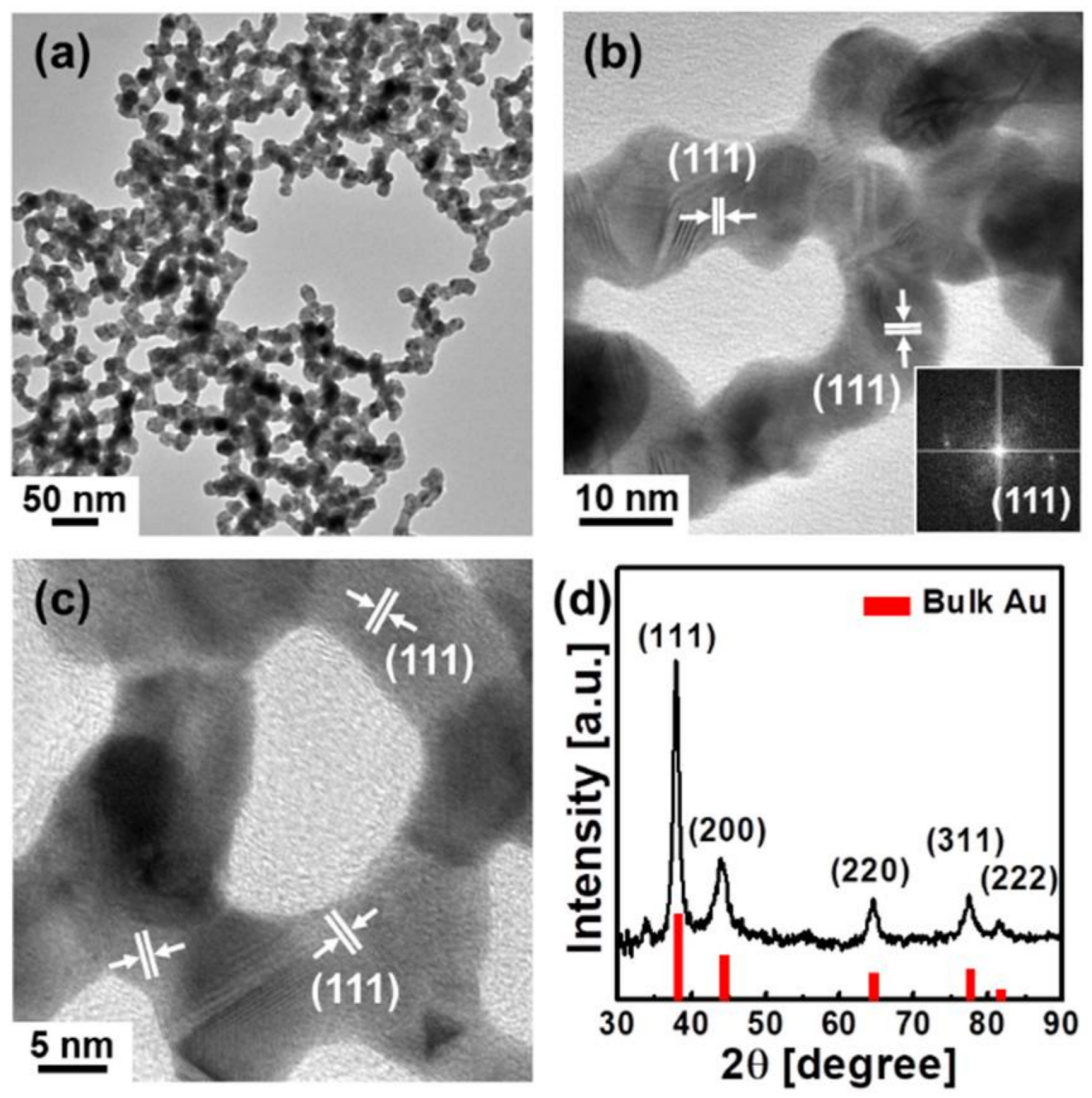
Characteristics of Au nanowire networks: (**a**,**b**) TEM images with different magnifications, the inset is the fast Fourier transform (FFT) image obtained from the panel (b); (**c**) HR-TEM images; (**d**) X-ray diffraction (XRD) pattern, red vertical lines indicate peak positions and relative intensities of bulk Au.

The Au nanowire networks showing absorption in the NIR regime were further investigated, as described below. The low-magnification TEM image of the Au nanowire networks in Figure 2a reveals that the wires have considerable lengths of a few micrometers. HR-TEM images of the Au nanowire networks are displayed in Figure 2b,c, indicating the dominant (111) lattice planes, in agreement with the fast Fourier transform (FFT) image shown in the inset. Moreover, these images provide insight into the crystallinity of the Au nanowire networks and their growth characteristics, acquired by the coalescence of Au particles and subsequent deposition of Au atoms, as suggested in previous studies [21,22,74].

The XRD pattern of the Au nanowire networks is presented in Figure 2d, showing that all characteristic peaks are indexed to the (111), (200), (220), (311) and (222) planes of the fcc Au crystal structure without any other phases, which also supports the TEM analysis.

When the solution containing the isolated Au nanowire networks was kept at room temperature for approximately one day, the color gradually changed from black to deep red, implying the transformation of the Au nanowire networks into spherical Au nanoparticles. The shape instability of nonspherical Au nanostructures has also been observed in previous works [21,22,42,43] and explained by the thermodynamic instability of the nonspheres [42,43]. To prevent the transformation of the Au nanowire networks, a stabilizing agent exhibiting stronger binding, the mPEG-SH ligand, was used. mPEG-SH stabilized the Au nanowire networks and remained intact without any color change over one month, as shown in Figure S1 in the Supplementary Materials. The absorption spectra and TEM image in Figure S1 also support that mPEG-SH coating stabilize Au nanowire networks without morphological change.

**Figure 3:**
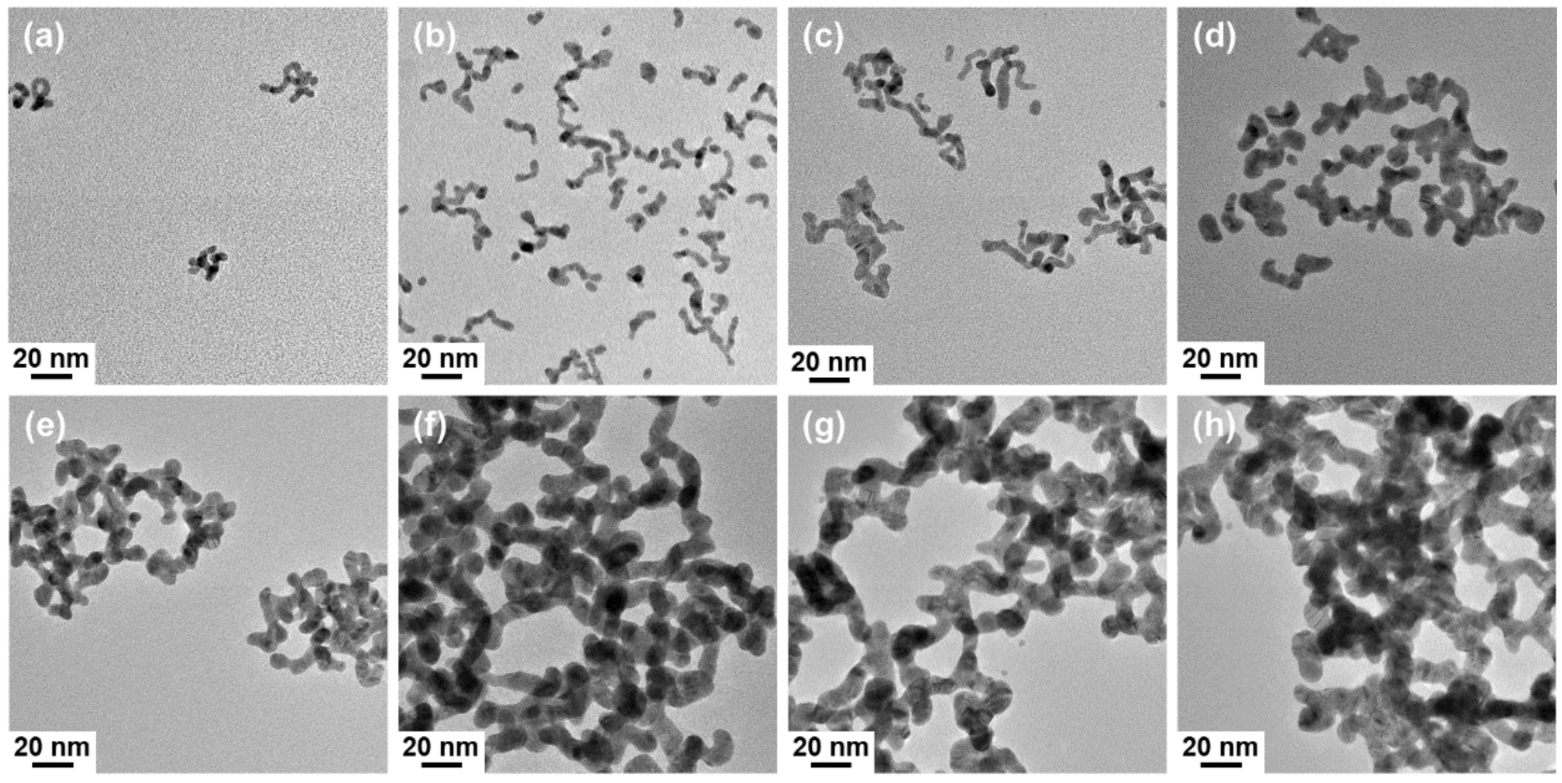
TEM images of the samples at different stages of the reaction: (**a**) 2 min; (**b**) 5 min; (**c**) 10 min; (**d**) 15 min; (**e**) 20 min; (**f**) 25 min; (**g**) 45 min; (**h**) 60 min.

To monitor the temporal evolution of the Au nanowire networks during the reaction time of 1 h, aliquots withdrawn at different reaction stages were analyzed, as shown in Figure 3. As seen in the TEM images, the length and diameter of the wires gradually increased as the reaction time progressed, implying that the Au nanowire networks were formed by depositing the Au monomer at the joints between the assembled Au nanocrystals. The absorption spectra of aliquots taken at different reaction stages are also shown in Figure S2, indicating that only one broad feature was seen at the beginning stages (e.g., 15 min). After 25 min, two distinct peaks were clearly observed, due to the TSPR and LSPR modes. This evolution shows that Au nanowire networks formed in nearly 25 min, in good agreement with the TEM analysis (see Figure 3f).

Several kinds of control experiments were performed to understand the effects of synthetic conditions. Without the addition of the EDTA tetrasodium salt, no Au nanostructures were formed. This supports the fact that the four carboxylic groups of the EDTA tetrasodium salt reduce the Au^3+^ species, which is in agreement with previous works [71,72,73]. It is noteworthy that EDTA tetrasodium salt introduced strong basic conditions, thus eliminating the need for additional steps (see Table S1 in the Supplementary Materials), whereas adjusting the pH by adding a suitable base was necessary for the preparation of the Au nanostructures in previous studies [72,75,76].

In addition, the addition of other EDTA salts was attempted for the synthesis of Au nanostructures. Under similar conditions such as temperature and concentration, the use of EDTA and EDTA disodium salt led to the formation of micrometer-sized and submicrometer-sized Au aggregates, respectively, as shown in Figure S3, revealing that the four deprotonated carboxyl groups (COO^−^) of EDTA tetrasodium salt as a tetradentate group are essential for allowing effective growth on the nanoscale.

NIR absorption is crucial for many biomedical applications because biological tissues, blood, and water show low absorption in this wavelength range [77,78]. The cytotoxicity of the Au nanowire networks coated with mPEG-SH was evaluated to demonstrate their applicability as photothermal therapeutic agents. The MTT cell proliferation assay is presented in Figure S4, indicating that more than 80% of the U87MG cells survived up to 1.27 mM (250 μg/mL) of the Au concentration. A more detailed procedure is presented in the Experimental Section.

**Figure 4:**
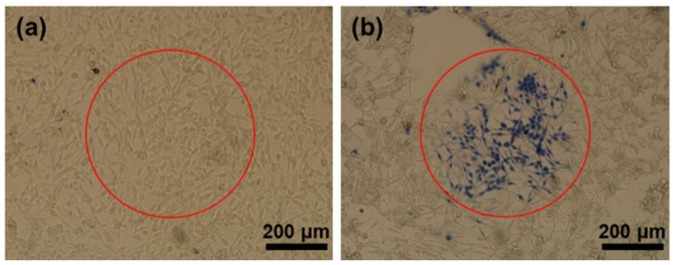
Optical microscope images of cancerous glioblastoma cells (U87MG) cells incubated (**a**) without; (**b**) with the Au nanowire networks, after irradiation for 10 min with a 980-nm near-infrared (NIR) CW diode laser and subsequent staining with 0.4% trypan blue.

Figure 4 represents the images of the U87MG cells without and with the Au nanowire networks after the NIR laser irradiation at 980 nm for 10 min, clearly revealing cell damage at the center of the laser beam for cells with an incubation of the Au nanowire networks, while the control cells remained intact. The dead cells were stained blue by 0.4% trypan blue. This observation clearly demonstrates the effectiveness of the Au nanowire networks in NIR photothermal therapy by selective killing of cancer cells, because of the local heating generated by their effective NIR absorption. Because of their relatively large size and irregular shape, the Au nanowire networks may not be suitable for animal study by systemic delivery. However, due to the good photothermal effect by NIR light, they can be used as a local heat generator to regulate cellular activity by attaching them to the cell membrane or biochip surface [79].

## 4. Conclusions

In summary, our work presents a facile and effective shape-controlled synthesis of Au nanostructures and their photothermal therapeutic effect. The described procedure involves the simple mixing of HAuCl_4_ and EDTA tetrasodium salt in an aqueous solution at room temperature, without additional ligands or toxic reagents. Adjusting the molar ratios of HAuCl_4_ to EDTA tetrasodium salt enables effective morphology control of Au nanostructures from spheres to branched forms and nanowire networks. Detailed control experiments revealed that the four deprotonated carboxylic acids of the EDTA tetrasodium salt provided effective growth control and stabilization. The Au nanowire networks showed strong absorption in the NIR region and hence were suitable for photothermal therapy. Under NIR irradiation, the Au nanowire networks allowed for selective destruction of cancerous U87MG cells by local heating, generated by the NIR absorption. This work demonstrates the development of a simple synthetic route to NIR-active Au nanostructures, which can be extended to other applications including optical sensing and surface-enhanced Raman scattering.

## Supplementary Materials

The following are available online at http://www.mdpi.com/2079-4991/8/4/252/s1, Figure S1: (a) Photograph of mPEG-SH stabilized Au nanowire networks kept in ambient conditions for one month, showing good stability, (b) absorption spectra of as-synthesized Au nanowire networks (black curve) and mPEG-SH coated Au nanowire networks (red curve), and (c) TEM image of Au nanowire networks after mPEG-SH coating, Figure S2: Absorption spectra of Au nanostructures taken at different stages of the reaction, Figure S3: TEM images of Au aggregates generated by using (a) EDTA and (b) EDTA disodium salt, Figure S4: The cell viability of Au nanowire networks by MTT assay, Table S1: The pH values at different reaction conditions.
