# Supplementary material for: Shape-Controlled Synthesis of Au Nanostructures Using EDTA Tetrasodium Salt and Their Photothermal Therapy Applications
Source: Nanomaterials (Basel). 2018 Apr 18;8(4):252. doi: 10.3390/nano8040252 (PMC5923582; doi:10.3390/nano8040252)
Supplement: Supplementary file 1 [file nanomaterials-08-00252-s001.pdf]

nanomaterials

Supplementary Materials

Shape-Controlled Synthesis of Au Nanostructures using EDTA Tetrasodium Salt and Their Photothermal Therapy Applications

Youngjin Jang [1], Nohyun Lee [2], Jeong Hyun Kim [3], Yong Il Park [4,*] and Yuanzhe Piao [5,*]

1 Schulich Faculty of Chemistry, Solid State Institute, Russell Berrie Nanotechnology Institute, Nancy and Stephen Grand Technion Energy Program, Technion–Israel Institute of Technology, Haifa 3200003, Israel; youngjin@technion.ac.il
2 School of Advanced Materials Engineering, Kookmin University, Seoul 02707, Republic of Korea; nohyunlee@kookmin.ac.kr
3 Center for Nanoparticle Research, Institute for Basic Science, and School of Chemical and Biological Engineering, Seoul National University, Seoul 151-742, Republic of Korea; jhkim113@snu.ac.kr
4 School of Chemical Engineering, Chonnam National University, Gwangju 61186, Republic of Korea
5 Graduate School of Convergence Science and Technology & Advanced Institutes of Convergence Technology, Seoul National University, Suwon 16229, Republic of Korea
* Correspondence: ypark@jnu.ac.kr (Y.I.P.); parkat9@snu.ac.kr (Y.P.); Tel.: +82-62-530-1886 (Y.I.P.); Tel: +82-31-888-9141 (Y.P.)

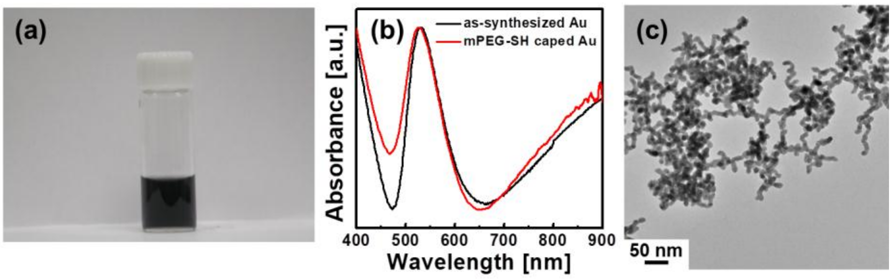

Figure S1. (a) Photograph of mPEG-SH stabilized Au nanowire networks kept in ambient conditions for one month, showing good stability, (b) absorption spectra of as-synthesized Au nanowire networks (black curve) and mPEG-SH coated Au nanowire networks (red curve), and (c) TEM image of Au nanowire networks after mPEG-SH coating.

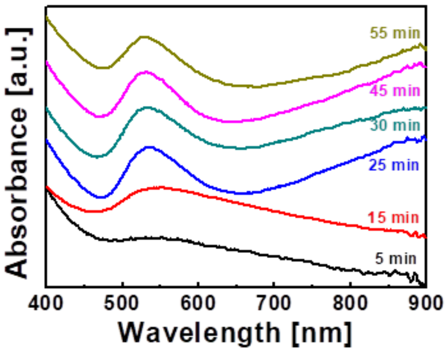

Figure S2. Absorption spectra of Au nanostructures taken at different stages of the reaction.

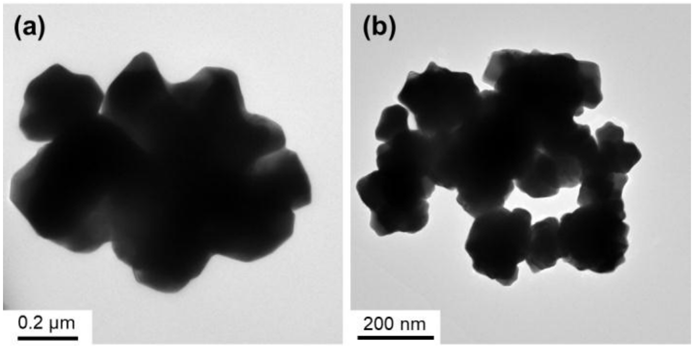

Figure S3. TEM images of Au aggregates generated by using (a) EDTA and (b) EDTA disodium salt.

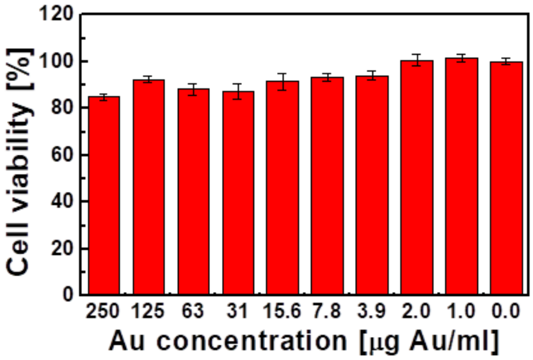

Figure S4. The cell viability of Au nanowire networks by MTT assay.

Table S1. The pH values at different reaction conditions.

Condition	pH
Aqueous solution containing 30 mg of EDTA tetrasodium salt in 7 mL of deionized water	10.98
Aqueous solution containing 30 mg of EDTA tetrasodium salt and 0.1 mL of 0.1 M $HAuCl_4$ in 7 mL of deionized water	9.77
Aqueous solution containing spherical Au nanocrystals after purification	8.81

© 2018 by the authors. Submitted for possible open access publication under the terms and conditions of the Creative Commons Attribution (CC BY) license (http://creativecommons.org/licenses/by/4.0/).
